# Double Trouble in a Patient with Ischemic Cardiomyopathy and Severe Mitral Regurgitation: A Case Report

**DOI:** 10.19102/icrm.2025.16061

**Published:** 2025-06-15

**Authors:** Ahmet Taha Sahin, Hasan Kan, Muhammet Fatih Kaleli, Ahmet Lutfu Sertdemir, Enes Elvin Gul

**Affiliations:** 1 Department of Cardiology, Beyhekim Training and Research Hospital, Konya, Turkey; 2 Department of Cardiology, Necmettin Erbakan University, School of Medicine, Konya, Turkey

**Keywords:** Atrial tachycardia, bidirectional ventricular tachycardia, coronary artery disease, dual tachycardia, mitral regurgitation

## Abstract

Bidirectional ventricular tachycardia (VT) is a rare arrhythmia characterized by alternating QRS morphologies and axis changes. Atrial flutter (AFL) can coexist with ventricular arrhythmias, complicating diagnosis. We present a case of a 56-year-old man with a history of ischemic heart disease and severe mitral regurgitation admitted with palpitations who was diagnosed with dual tachycardia (bidirectional VT and AFL).

## Introduction

Bidirectional ventricular tachycardia (VT) is a rare arrhythmia with a distinct electrocardiographic (ECG) pattern, characterized by alternating QRS morphologies and axis changes.^1^ It is most commonly associated with digoxin toxicity, but other etiologies such as catecholaminergic polymorphic VT (CPVT), myocardial infarction, myocarditis, aconitine poisoning, and Andersen–Tawil syndrome have been reported.^2^ Additionally, atrial flutter (AFL) can coexist with or mimic other arrhythmias, complicating diagnosis and management.^3^ Combined bidirectional VT and AFL is exceptionally rare, particularly in patients with ischemic heart disease (IHD) and valvular pathologies. Here, we present a unique case of dual tachycardia—bidirectional VT and AFL—in a patient with severe mitral regurgitation (MR), moderate tricuspid regurgitation (TR), and impaired left ventricular function, which was managed with an electrophysiological (EP) study (EPS) and heart team intervention.

## Case presentation

**Figure 1:**
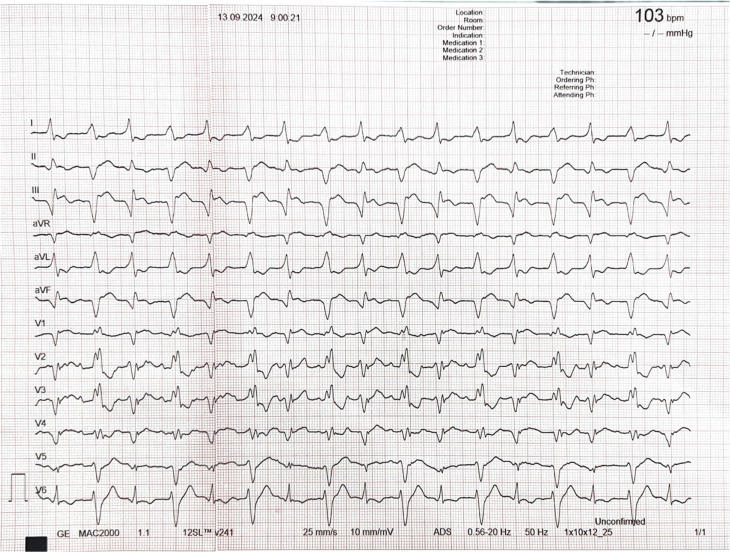
A baseline 12-lead electrocardiogram recorded on admission showing wide complex tachycardia with alternating QRS morphologies and axis change.

A 56-year-old man presented to the emergency room with palpitations and shortness of breath. He had a history of IHD but no prior arrhythmic events or sudden cardiac death in his family. On admission, the patient was hemodynamically stable. The initial 12-lead ECG revealed a wide QRS complex tachycardia with alternating QRS morphologies (right and left bundle branch block [LBBB]) and axis changes. The heart rate was 103 bpm. Despite clearly visible P-waves in the precordial leads, there was no apparent relationship between atrial and ventricular activity, suggesting atrioventricular (AV) dissociation **(Figure 1)**.

Transthoracic echocardiography demonstrated an impaired left ventricular ejection fraction of 40%, an enlarged left atrium (56 mm), severe MR, and moderate TR. Based on the clinical findings, the patient was admitted to the coronary care unit.

Initial differential diagnoses included VT and AFL with aberrancy in a bigeminy pattern. Intravenous adenosine (12 mg) was administered to assess atrial tachycardia (AT) with aberrancy; however, this did not alter the heart rate or QRS morphology, ruling out AT with aberrancy. The patient was subsequently taken to the EP laboratory for further investigation.

**Figure 2:**
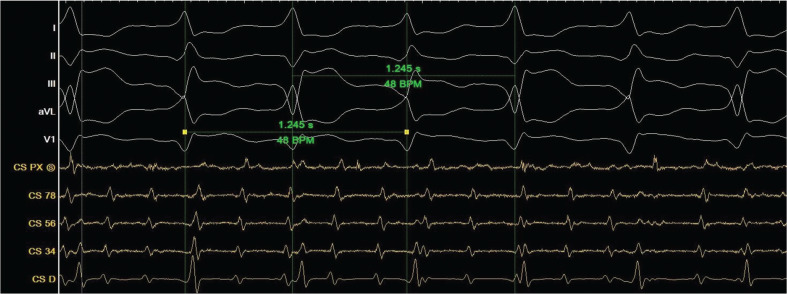
An electrophysiological study showing both atrial tachycardia and bidirectional ventricular tachycardia. The cycle length between two different QRS morphologies was identical at a rate of 1245 ms. Heart rate, 96 bpm.

The insertion of a coronary sinus catheter revealed simultaneous AFL and bidirectional VT **(Figure 2)**. Electrical cardioversion restored sinus rhythm, but subsequent rhythm monitoring revealed slow VT with AV dissociation and intermittent sinus beats. After careful interpretation of both the ECG and electrograms, a final diagnosis of dual tachycardia of bidirectional VT and AFL was made.

The patient was presented at a multidisciplinary heart team meeting. Given the structural heart disease and severe valvular abnormalities, the team recommended mitral valve replacement and tricuspid valve repair. The patient underwent surgery and had an uneventful recovery.

## Discussion

Bidirectional VT is a rare and intriguing arrhythmia, most often associated with digitalis toxicity.^4^ Less common causes include CPVT, Andersen–Tawil syndrome, aconitine poisoning, myocarditis, hypokalemic periodic paralysis, and myocardial infarction.^5^ It is defined electrocardiographically by alternating QRS morphologies and a regularly irregular rate. The alternating pattern is believed to result from automaticity or re-entry mechanisms involving distinct conduction pathways.

In this case, the patient had no history of digoxin use, catecholaminergic disorders, or family history of sudden cardiac death, making coronary artery disease (CAD) with associated structural abnormalities the most plausible underlying cause. Severe MR and moderate TR, with resultant atrial dilation and ventricular strain, likely contributed to the arrhythmic substrate. The presence of dual tachycardia further complicated the diagnosis and management, as the interaction between bidirectional VT and AFL with aberrancy is poorly understood.

The complexity of dual tachycardia, particularly involving bidirectional VT, has been explored in several case studies. For instance, Waroux et al.^6^ reported a case of wide QRS complex tachycardia with group beating in a young patient with heart failure. Their study emphasized the importance of a detailed EP evaluation to identify underlying mechanisms, highlighting how group beating can result from re-entry or competing automatic foci. Similarly, in our case, an EPS was pivotal in identifying the simultaneous presence of bidirectional VT and AFL, allowing for accurate diagnosis and guiding management.

In the study by Baranchuk et al.,^7^ grouped beats were analyzed in the context of bradycardia-dependent LBBB and pseudo-supernormal conduction. They described cases where changes in conduction velocity mimicked alternating arrhythmias. However, pseudo-supernormal conduction does not explain the alternating QRS morphology seen in bidirectional VT, as the QRS alternation in our patient was unrelated to conduction delays. This distinction is critical, as the mechanism underlying pseudo-supernormal conduction is often misinterpreted in similar cases.

Bidirectional VT arises from alternating ventricular activation, often involving distinct Purkinje fibers or two automatic foci. Structural abnormalities, such as severe MR and atrial dilation, can increase myocardial irritability and predispose to arrhythmias. In our case, the concurrent presence of AFL further complicated the clinical picture. The misinterpretation of bidirectional VT as pseudo-supernormal conduction or aberrancy, as highlighted in Baranchuk et al.,^7^ is a potential pitfall, underscoring the value of an EPS in clarifying the mechanism.

Royle et al.^8^ described a case of AT with group beating and explored the interplay between atrial and ventricular arrhythmias. Their findings underscored the diagnostic complexity when arrhythmias coexist, requiring advanced EP evaluation to delineate the distinct arrhythmias. Our case parallels this diagnostic challenge, as the patient exhibited simultaneous bidirectional VT and AFL, initially misinterpreted as a single arrhythmic mechanism.

The differential diagnosis of bidirectional VT must include a thorough evaluation of the patient’s medication history, genetic predispositions, and electrolyte imbalances. EPS remains the gold standard for diagnosing complex arrhythmias, as demonstrated by the identification of dual tachycardia in this patient.

## Conclusion

This case represents a unique instance of dual tachycardia—bidirectional VT and AF—in a patient with IHD, severe MR, and moderate TR. The interplay between structural heart disease and arrhythmias underscores the complexity of such cases and the importance of a multidisciplinary approach. To the best of our knowledge, this is the first reported case of combined bidirectional VT and AFL in a patient with CAD and valvular dysfunction. Further research is needed to understand the mechanisms and optimal management of such rare arrhythmias.
